# Single trial prestimulus oscillations predict perception of the sound-induced flash illusion

**DOI:** 10.1038/s41598-019-42380-x

**Published:** 2019-04-12

**Authors:** Mathis Kaiser, Daniel Senkowski, Niko A. Busch, Johanna Balz, Julian Keil

**Affiliations:** 1 Department of Psychiatry and Psychotherapy, Charité–Universitätsmedizin Berlin, Große Hamburger Str. 5-11, 10115 Berlin, Germany; 2 Berlin School of Mind and Brain, Humboldt-Universität zu Berlin, Luisenstraße 56, 10117 Berlin, Germany; 3 Institute of Psychology, University of Münster, Fliednerstr. 21, 48149 Münster, Germany; 4 Biological Psychology, Christian-Albrechts-University Kiel, Olshausenstraße 62, 24118 Kiel, Germany

## Abstract

In the sound-induced flash illusion, auditory input affects the perception of visual stimuli with a large inter- and intraindividual variability. Crossmodal influence in this illusion has been shown to be associated with activity in visual and temporal areas. In this electroencephalography study, we investigated the relationship between oscillatory brain activity prior to stimulus presentation and subsequent perception of the illusion on the level of single trials. Using logistic regression, we modeled the perceptual outcome dependent on oscillatory power. We found that 25 Hz to 41 Hz activity over occipital electrodes from 0.17 s to 0.05 s prior to stimulus onset predicted the perception of the illusion. A t-test of power values, averaged over the significant cluster, between illusion and no-illusion trials showed higher power in illusion trials, corroborating the modeling result. We conclude that the observed power modulation predisposes the integration of audiovisual signals, providing further evidence for the governing role of prestimulus brain oscillations in multisensory perception.

## Introduction

In order to successfully navigate our environment, it is vitally important to integrate information from various sensory sources. Recent studies have highlighted the crossmodal influence between sensory modalities and the underlying neural mechanisms^1^. An established paradigm to study crossmodal influence is the sound-induced flash illusion (SIFI), where auditory input affects the processing and perception of visual input. In a typical SIFI paradigm, participants are presented with one or more brief sound stimuli concurrently with one or more bright visual stimuli. Participants are then asked to report the number of perceived flashes. The illusion manifests itself in responses that are influenced by auditory information; the number of flashes that participants subjectively perceive depends on the number of sounds they have heard^2^. We here regard occurrence of the SIFI as an example of multisensory integration resulting from crossmodal stimulation^3^. Importantly, the illusion does not occur in all trials, but with a large intra- and interindividual variability^4^. This allows for analysis approaches that differentiate the neural activity patterns that predict the perceptual events of interest.

With regard to the neuroanatomical substrates of crossmodal influence in the SIFI, activity in occipital and temporal areas has been shown to be associated with illusory flash perception: Studies using functional magnetic resonance imaging (fMRI) have established that the activity level in V1 reflects the number of flashes subjectively perceived, with increased activity for additionally perceived flashes and decreased activity for perceptual fusion, i.e. perception of one flash following presentation of two flashes and one sound^5,6^. Interestingly, activity in the right superior temporal gyrus is increased in both cases. This suggests that activity in lower and higher tier areas differentially reflects the processing and integration of multisensory stimuli. Consistent with these findings, event-related potentials (ERPs) over occipital areas, as obtained through electroencephalography (EEG), are modulated around 100 ms after stimulus onset in the SIFI^7,8^. Moreover, early gamma power over occipital areas is increased following the illusion^9,10^, and the mid-latency gamma power increase in temporal areas is correlated with increased likelihood of perceiving the illusion^11^. While early modulations of ERPs and spectral activity in electrophysiological studies have traditionally been viewed as correlates of modality-specific processing in unisensory areas, influences from other unisensory, polymodal and frontal areas cannot be ruled out^12,13^.

In recent years, the brain states and network configurations predisposing multisensory integration and – more generally – conscious perception have come into focus^14–16^. Several studies have established links between oscillatory brain activity prior to stimulus onset and subjective perception of multisensory stimuli^17–19^. Using magnetoencephalography (MEG), Keil *et al*.^18^ have found that beta band power is increased in the left temporal gyrus prior to perceiving the SIFI. Moreover, in a visuotactile flash illusion paradigm, Lange *et al*.^19^ have found that decreased alpha band power in occipital areas and increased gamma band power in occipitoparietal and right temporal areas precede illusory flashes. These studies provide the primary research background for the current study. However, these studies analyzed averaged estimates of oscillatory power across trials, thereby neglecting the trial-by-trial fluctuations of cortical activity, which are potentially informative regarding variability in perception and other cognitive functions^20,21^.

Here, we aimed to elucidate the relationship between oscillatory brain activity prior to stimulus presentation and perception of the SIFI on the level of single trials, initially focusing on the frequencies up to 41 Hz to follow previous analysis protocols^18^ and then extending to higher frequencies. We employed a logistic regression model that predicts the binary perceptual outcome (*illusion*/*no illusion*) dependent on the level of oscillatory activity in each trial. By incorporating single trial information in the analysis, we aimed to detect subtle modulations of oscillatory activity that might go unnoticed when averaged power is statistically compared between different perceptual outcomes. When trial numbers are strongly unbalanced across conditions, trial numbers are often equalized in conventional analyses by random sampling, thereby losing information. Employing a regression model allowed us to circumvent this. Based on the previous work, we hypothesized that ongoing oscillatory activity influences upcoming perception on the trial-by-trial level. We also explored a possible relationship between prestimulus activity and early evoked potentials by correlating prestimulus oscillatory power differences with previously demonstrated ERP amplitude differences^10,22^.

## Results

**Figure 1:**
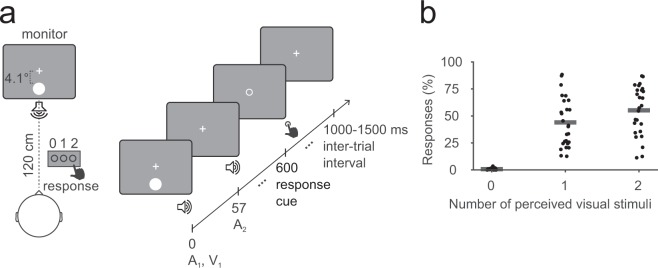
(**a**) Experimental design. (**b**) Response rates in the A_2_V_1_ condition. Individual response rates are indicated by dots, the average is indicated by horizontal bars.

In this experiment, six audiovisual stimulus combinations were presented: A_0_V_1_, A_0_V_2_, A_1_V_1_, A_2_V_0_, A_2_V_1_ and A_2_V_2_, where the indexed numbers represent the number of auditory (A) and visual (V) stimuli. A_2_V_1_ is the illusion condition, where either one or two flashes can be perceived. Illusion rates in the sample varied between 11 and 87% (mean: 55 ± 22% SD, also see Fig. 1b). Behavioral accuracies (referring to veridical visual perception) in the conditions were as follows (mean ± SD): A_0_V_1_: 83 ± 15%, A_0_V_2_: 83 ± 16%, A_1_V_1_: 94 ± 9%, A_2_V_0_: 92 ± 9%, A_2_V_1_: 44 ± 22%, A_2_V_2_: 93 ± 12%. Accuracies above 80% in all but the illusion condition (A_2_V_1_) indicate that subjects generally responded accurately to visual input.

**Figure 2:**
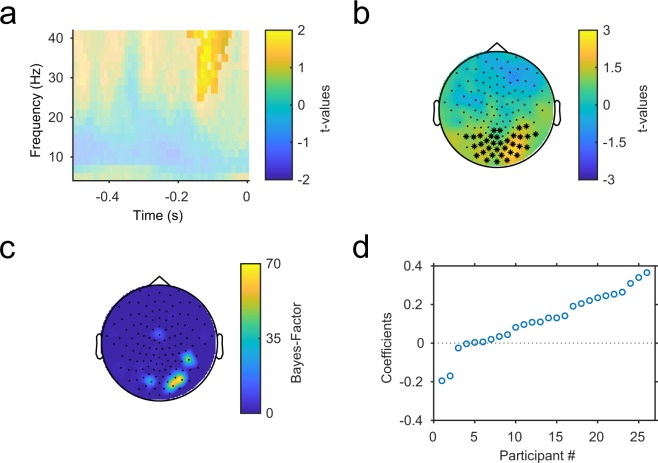
(**a**) Time frequency spectrum of t-values for the comparison of observed regression weights against dummy data, averaged over the significant channels. Significant regions are indicated by saturation. (**b**) Topography of t-values, averaged over the significant time-frequency region. Significant channels are marked by asterisks. (**c**) Topography of Bayes-Factor values, averaged over the significant time-frequency region. (**d**) Individual regression coefficients, averaged over the significant cluster. Participants are sorted in ascending order of the coefficient.

When quantifying the relationship between single-trial EEG power in the prestimulus time window and behavioral outcome, we found a significant cluster of regression weights over occipital electrodes between 25 and 41 Hz, i.e. the high beta to low gamma band, from 0.17 s to 0.05 s before stimulus onset (*p* = 0.006, Fig. 2a,b). The individual regression coefficients, averaged across the cluster, were positive in 22 of 26 participants, indicating a positive relationship between increased beta/gamma band power and SIFI perception (Fig. 2d). We found high Bayes Factor values at a subset of right occipitotemporal electrodes, indicating strong support for the hypothesis that oscillatory power at these electrodes predicts perception on a trial-by-trial basis (Fig. 2c). The mean of Bayes factor values, averaged across the cluster, was 67.61. A dependent-samples t-test of normalized power values averaged over the significant channel/frequency/time window (*illusion* vs. *no illusion*) resulted in a significant effect (*t*(25) = 3.13, *p* = 0.0045). This further supports the notion that oscillatory power has predictive value for crossmodal influence. An unrestricted, cluster-corrected dependent-samples t-test for the same comparison did not reveal significant effects (*p* = 0.51 for the positive cluster with the largest effect and comparable extent to the modeling result). This indicates that the single-trial modeling approach can uncover relationships that are not picked up by conventional analyses comparing means between conditions.

Since the significant effect was located at the upper end of the analyzed frequency window, we additionally performed an analysis of gamma activity (40 to 100 Hz), which did not reveal any significant effects. The cluster with the largest effect in the gamma band extended from 40 to 45 Hz and from 0.13 to 0.05 s prior to stimulus onset (*p* = 0.1018).

Using a cluster-corrected dependent-samples t-test, we replicated previous findings^10,22^ of an ERP amplitude difference over central sensors in the time range of 0.1 to 0.17 s after stimulus onset (*p* = 0.004, see supplementary Fig. S1). Amplitudes of this early negative component were larger in *illusion* compared to *no illusion* trials. To link pre- and poststimulus activity, we calculated a Pearson’s correlation between the identified differences in oscillatory power and ERP amplitudes across participants. However, the correlation did not yield a significant result (*r* = 0.16, *p* = 0.43, see supplementary Fig. S1).

## Discussion

In this study, we investigated the relationship between single-trial prestimulus oscillatory brain activity and audiovisual crossmodal influence in the sound-induced flash illusion using a logistic regression model. Across participants, increased power over occipital electrodes between 25 and 41 Hz, starting 170 ms before stimulus onset, predicted subsequent perception of the illusion. Although the effect was located at the upper end of the analyzed frequency window, a subsequent analysis of high-frequency gamma band power resulted in no significant effects. There was a trend level cluster in the gamma analysis that could be part of the cluster obtained in the lower-frequency analysis. However, since that cluster extended only to 45 Hz, this would not substantially change the conclusion that the contribution of oscillatory power to illusory perception seems to be restricted to the upper beta/lower gamma frequency range. In a follow-up analysis, we calculated Bayes-Factor values for the t-values resulting from the comparison of regression weights against random data with the same mean and standard deviation, which quantify the empirical support for the alternative hypothesis (i.e. that the regression weights are significantly different from noise). We found the most robust effects over right occipitotemporal channels, suggesting a contribution of occipital and, possibly, right temporal areas to the observed effect, although this suggestion must remain speculative at this point since no source analysis was performed. The exploratory analysis of a relationship between prestimulus power and ERP amplitude differences did not reveal a significant effect. It is worth noting that this does not preclude a link between prestimulus oscillatory activity and other types of stimulus-induced responses.

Previous studies investigating prestimulus oscillatory activity in double flash illusions using MEG have found that crossmodal influence is associated with increased beta band power (13–21 Hz) in left temporal areas^18^; enhanced excitability in visual areas, as reflected by decreased alpha band power; and increased gamma band power in a more extensive cortical network including temporal areas^19^. Prestimulus alpha band power is also decreased in the triple flash illusion, where two rapidly presented flashes are occasionally perceived as three^23^. Interestingly, the optimal delay between flashes is correlated with the subject-specific impulse response frequency, pointing to a contribution of oscillatory reverberation to illusory visual perception. Similarly, the phase of ongoing alpha band oscillations influences crossmodal synchrony perception^24^. A phase reset of ongoing slow oscillations in unisensory areas is a likely mechanism for the modulation of activity following heteromodal input^13,25,26^. The ensuing phase alignment increases local, modality-specific excitability and promotes efficient communication between different cortical areas. However, prestimulus alpha band power did not have predictive value for perception of the SIFI in the present study, and analyses of the possible influence of oscillatory phase were beyond scope.

Previously, Lange *et al*.^19^ reported that a prestimulus modulation of excitability in occipital and temporal areas, as reflected by decreased alpha and increased gamma band power, entails perception of the SIFI. This modulation of excitability might facilitate the crossmodal influence of auditory input on visual stimulus processing. Although the frequency range of the currently observed effect is lower than the gamma band effect previously reported, the functional roles might be similar. Interestingly, a recent study combining EEG and Bayesian modeling of behavioral responses in a SIFI paradigm has found a correlation between prestimulus oscillatory gamma activity over occipital electrodes and the perceptual prior that signals originate from a common source^27^. In other words, high prestimulus occipital gamma power increased the tendency to perceptually bind audiovisual signals in the SIFI. The spectrotemporal extent of the highest correlation values has a substantial overlap with the significant cluster in the present study.

A study using transcranial direct current stimulation (tDCS) has shown that up-regulation of occipital cortex results in decreased illusion rates in the SIFI, while up-regulation of temporal cortex results in increased illusion rates^28^. Furthermore, the detection of TMS-induced phosphenes, which are enhanced by concurrent auditory or tactile stimulation, selectively benefits from tDCS over temporal and parietal, but not occipital cortex^29^. These findings suggest that variations in crossmodal influence are a function of excitability in the cortical areas where influence originates, rather than in the target sites of influence. A possible reason for this is that excitability changes induced by tDCS promote unisensory processing in the target sites. Related to this notion, inferring the spatial location of incongruent audiovisual signals is performed by a hierarchy of cortical areas: primary visual and auditory areas estimate location under the assumption of independent sources, while more parietal areas represent location under the assumption of a common source and integrate the estimates, which results in crossmodal influence^30^. This means that, although functional interactions and direct projections between heteromodal unisensory areas have been demonstrated^25,31^, the representation resulting from weighting of perceptual priors and sensory input seems to rely more on multisensory than unisensory areas in the case of crossmodal influence. Given the context of these findings, the predictive value of high-beta/low-gamma band oscillations for the SIFI that we found in the present study might capture a biasing influence of auditory information on multisensory integration areas (e.g. superior temporal gyrus). Related to this, a previous study showed that increased lower beta band functional connectivity between auditory and multisensory temporal areas but decreased connectivity between temporal and visual areas is related to the perception of the SIFI^18^. Studies investigating the mechanisms of interareal information transfer in the visual system have shown that the direction of influence is correlated with activity in distinct frequency bands: feedforward influence is carried by gamma band synchronization, and feedback influence is carried by beta band synchronization^32,33^. Furthermore, top-down influence in the beta band has been shown to enhance bottom-up gamma band responses around 100 ms later^34^. We therefore suggest that increased 25 Hz to 41 Hz oscillatory power that predicts perception of the SIFI in the present study could reflect the interplay of feedback and feedforward influence between auditory, visual and multisensory regions. Nevertheless, more conclusive evidence for this suggestion, as well as for the direction of influence and the importance of local excitability, should be obtained in future studies employing directed connectivity metrics on the source level.

Given reports that crossmodal interaction in the SIFI is associated with increased gamma band power after stimulus onset^9–11^, one could argue that the current results are confounded with spectral leakage from the post-stimulus period. The time window we used for time frequency analysis is 3 cycles at each frequency, resulting in a time window of 0.12 s at 25 Hz (shorter for increasing frequencies). Hence, 25 Hz activity from up to 0.06 s later may have leaked into a given data point being analyzed (albeit less towards the edge of the time window due to tapering, and even less for higher frequencies). It is unlikely that the effect we found, which extended from 25 to 41 Hz and from 0.170 to 0.05 s prestimulus, was substantially affected by poststimulus activity, since it already arises long before substantial contamination may have occurred: only the last 0.01 seconds at the lower frequency edge of the cluster may have been affected. Another concern may be our non-canonical analysis approach, consisting of a logistic regression of a binary perceptual outcome on oscillatory activity and a subsequent test of the obtained regression weights against random noise. Since such an analysis has not been applied to EEG data in this manner before, we validated the finding by performing a classical t-test of power values averaged over trials of the two perceptual conditions, which confirmed that power was higher in *illusion* vs. *no illusion* trials. We therefore argue that our approach, although it requires further validation, merits application because it allows modeling perceptual variation on the single trial level, and resulted in findings that could be confirmed using more established methods.

Future research could address the role of crossmodal phase resetting in the SIFI and the direction of information transfer between relevant brain areas. Such studies would also allow insight into the relative importance of local excitability changes in sensory areas and feedback by higher-order areas^1^. Furthermore, the sources of modulations in oscillatory activity should be localized more accurately to characterize the pertinent network topology and corresponding activity patterns. Electrocorticography is one method that has been successfully employed to study the mechanisms of crossmodal influence with sufficiently high temporal and spatial resolution^26^. A limitation of the current study is that it is unclear whether the effect of prestimulus oscillations specifically affects the SIFI, i.e. the number of illusory flashes in the A_2_V_1_ condition. Instead, prestimulus oscillations might generally affect accuracy or bias in the flash-counting task irrespective of the veridical number of flashes. However, modeling the perceptual outcome in the other conditions was not feasible due to ceiling effects. In follow-up studies, it would therefore be interesting to modify stimuli such that conditions with two visual flashes and with no visual flashes (A_2_V_2_ and A_2_V_0_, respectively) yield greater variability in the number of reported flashes, and to include the veridical number of flashes as an additional predictor in the regression model. It would then be possible to differentiate the influence of visual input, prestimulus oscillations, and their interaction.

To conclude, the present study demonstrates that single-trial oscillatory activity predicts the integration of multisensory signals, as exemplified by an influence of auditory input on visual perception in the SIFI. A number of studies have found early modulations of cortical activity over visual areas related to the SIFI, which can be integrated in the context of the present findings: We propose that crossmodal influence in the SIFI is facilitated already before stimulus onset. This facilitation is reflected by reduced alpha band power in occipital areas and increased gamma band power in temporal areas^19^, which might indicate enhanced excitability of polysensory pathways. Changes in excitability induced in visual cortex by tDCS, which result in changes of the illusion rate^28^, are likely confined to unisensory visual pathways. Furthermore, multisensory areas in the superior temporal gyrus, as well as frontal areas, exert influence over audiovisual information flow, which is reflected by modulation of beta band oscillations^18^. These brain areas, which are relevant for a predisposition for crossmodal influence, are also involved in stimulus processing: activation levels in early visual areas indicate the number of subjectively perceived stimuli^5,8^, while activity in superior temporal areas reflects crossmodal interaction more generally^6,11^. Importantly, these later modulations of cortical activity may already be primed by the ongoing prestimulus oscillations revealed in the present study.

## Methods

### Participants

Forty healthy volunteers participated in the study. All participants had normal hearing and normal or corrected-to-normal vision, and reported no history of neurological or psychiatric disorders. All participants gave written informed consent, the study was conducted in accordance with the Declaration of Helsinki and approved by the ethics committee of the Charité–Universitätsmedizin Berlin. Findings from this dataset focusing on independent aspects of neural processing have previously been reported^4,11,22^. In accordance with our previous analysis of alpha frequency from the same dataset, a subset of 26 participants who showed illusion rates between 10 and 90% were selected for analysis^4^. The illusion rate is computed as the proportion of trials containing two auditory stimuli and one visual stimulus (A_2_V_1_) where there is an illusory perception of two flashes. The reason for excluding participants with extreme illusion rates is that these participants either did not reliably perceive the illusion, or primarily relied on auditory instead of visual input in reporting the number of perceived flashes. The mean age of the 26 selected participants (8 female; 1 left-handed) was 33.7 years (range: 18–51 years).

### Experimental design

The experiment was conducted in a sound-attenuated electrically shielded chamber. Visual stimuli were presented on a CRT monitor with a background luminance of 21 cd/m^2^ and a refresh rate of 75 Hz. Auditory stimuli were presented from a central speaker below the screen. Six stimulus combinations were presented: A_0_V_1_, A_0_V_2_, A_1_V_1_, A_2_V_0_, A_2_V_1_ and A_2_V_2_, where the indexed numbers represent the number of auditory (A) and visual (V) stimuli. A_2_V_1_ is the illusion condition, while the other combinations served as behavioral control trials. We did not use all possible stimulus combinations due to time constraints. Visual stimuli were presented for 10 ms and consisted of a white disk subtending a visual angle of 1.6° with a luminance of 89 cd/m^2^. Visual stimuli were presented at 4.1° centrally below the fixation cross. Auditory stimuli were presented for 7 ms and consisted of a 73 dB (SPL) 1000 Hz sine wave tone. Three hundred A_2_V_1_ trials and 150 trials per control condition were presented in random order in eight blocks. Subjects were asked to indicate how many visual stimuli they perceived (zero, one or two) with a button press of their right hand. For details of the experimental setup, see Fig. 1a.

### Acquisition and Preprocessing of EEG data

EEG was recorded using a 128-channel active electrode cap (EasyCap, Herrsching, Germany), including one horizontal and one vertical electrooculography electrode to monitor eye movements, and Brainamp DC amplifiers (Brainproducts, Gilching, Germany). Data were recorded in reference to an electrode placed on the nose with a sampling frequency of 1000 Hz and a pass band from 0.016 to 250 Hz.

EEG data processing was performed in MATLAB (MathWorks, Natick, MA, USA) using the EEGlab^35^ and FieldTrip^36^ toolboxes and custom scripts. Data were filtered using a two-pass Hamming-windowed FIR filter, with an order of 2999 and a −6 dB cutoff frequency of 1 Hz for the high pass, and an order of 23 and a cutoff frequency of 125 Hz for the low pass. A 4th-order two-pass Butterworth filter with a stop band from 49 to 51 Hz was used to filter out line noise. Data were subsequently downsampled to 500 Hz and epoched from −1 to 3 s relative to the first auditory stimulus onset. Trials and channels that contained large artifacts were removed after visual inspection. Independent component analysis using an extended infomax algorithm^37^ was performed on the truncated data and components that represented eye blinks or cardiac activity were removed. Electrooculography channels were removed from the data and rejected EEG channels were interpolated using spherical interpolation. Next, data were re-referenced to common average. Trials that still exceeded a threshold of ±100 µV after these procedures were rejected automatically. On average, 98.65 (±50.9 SD) trials, 1.35 (±1.39 SD) channels, and 15.65 (±6.7 SD) components were removed from each dataset. Finally, epochs were sorted according to combination of audiovisual stimuli and response and A_2_V_1_-trials, where 1 (*no illusion*) or 2 flashes (*illusion*) were perceived, were selected. After preprocessing, there were on average 144.65 (±59.11 SD) A_2_V_1_ -trials, in which an illusion was perceived, and 116.35 (±61.82) A_2_V_1_-trials, in which no illusion was perceived in the individual datasets.

### Analysis of EEG data

Single trial EEG data were time-frequency transformed using a single Hanning taper with a window length of 3 cycles at each frequency. Time-frequency analysis was performed for the time window from −1 to 1 s around the onset of the first auditory stimulus, with a step size of 10 ms, for the frequencies from 5 to 41 Hz, with a frequency resolution of 2 Hz. We additionally performed an analysis of activity in the gamma frequency band from 40 to 100 Hz in steps of 5 Hz, using multiple tapers with a window length of 200 ms and frequency smoothing of ±10 Hz. The only difference in the analysis protocols for the lower and higher frequencies was the use of single and multiple tapers, respectively. We have used multiple tapers for higher frequencies because they offer better control over temporal and spectral resolution, and thus better signal-to-noise ratio for higher frequencies. Statistical modeling was otherwise identical. Thus, the analysis for higher frequencies can be considered an extension of the primary analysis. Taken together, they should reveal the entire frequency range of the observed effect.

To quantify the relationship between single-trial EEG power in the prestimulus time window and behavioral outcome, we calculated logistic regression weights between the spectral activity in a 500 ms time window prior to stimulus presentation and the binary perceptual rating (i.e. *illusion* vs. *no illusion*) separately for each electrode, frequency, and time-point. Before calculation of the regression weights, time-frequency data were scaled between zero and one, and then the inverse of the normal cumulative distribution function was taken. This was done in order to approximate the data to a normal distribution due to concerns regarding the use of regression approaches with non-normal distributed data^38^. The regression model can be stated as follows:

$$\mathrm{log}(\frac{{p}_{illusion}}{{\rm{1}}-{p}_{illusion}})={\beta }_{0}+{\beta }_{1}X$$

where *p*_*illusion*_ is the probability of perceiving the illusion, *β*_0_ is the intercept, *β*_1_ is the regression weight, and X is the normalized single-trial power.

For statistical evaluation, a cluster-based permutation test was used^39^. Within each subject, we calculated the mean and standard deviation of the observed regression weights across channels, frequencies and time-points. We then generated dummy regression weights for each subject that were randomly selected from a normal distribution with the mean and standard deviation calculated in the first step. For the group-level analysis, we compared the observed regression weights against these dummy data using a dependent-samples t-test with cluster correction. Samples at a given channel/frequency/time-point entered a cluster when the significance level exceeded 0.05 in at least three neighboring electrodes. The test statistic was computed as the sum of t-values within a cluster. The comparison was repeated with permuted condition labels 1000 times, yielding a distribution of test statistics under the null hypothesis. The significance level of the cluster was computed as the proportion of permutations that resulted in a test statistic exceeding the observed one. The aim of this procedure was to identify clusters of regression weights that stand out from the subject-specific distribution. Instead of testing the null hypothesis that the population level regression weights are zero, this procedure makes no assumptions about the real distribution of coefficients, which might be negatively or positively biased for single subjects. While this approach does not allow the identification of clusters of coefficients with high or low absolute values, it does detects those clusters of coefficients that differ from the subject-specific distribution. Based on the absolute regression weights within the cluster, we assessed the direction of the relationship between power and perception.

Additionally, within the channel/frequency/time cluster identified above, we performed a conventional t-test of normalized power values averaged over different perceptual conditions (*illusion* vs. *no illusion*) to check the robustness of the model results. We restricted this analysis to the time/frequencies/channels derived from the regression analysis to corroborate the result within the observed cluster. We performed an unrestricted cluster-based dependent samples t-test (*illusion* vs. *no illusion*) with the same parameters as in the statistical test for the regression weights to examine whether the modeling approach is more sensitive than conventional methods. We also calculated the Bayes factor (BF) based on the outcome of a dependent-samples t-test between observed regression weights and dummy data. The BF summarizes the ratio of evidence for a true effect vs. the evidence for the null hypothesis of no effect and was computed from the t-values using the *ttest*.*tstat* function from the BayesFactor R package^40^.

To explore whether differences in prestimulus oscillatory power affect differences of ERP amplitudes related to different perceptual outcomes, we calculated a Pearson’s correlation between both measures across participants. We first calculated ERPs for *illusion* and *no illusion* trials separately, applying baseline-correction with the 200 ms interval before stimulus onset and a 30 Hz low-pass filter (hamming-windowed sinc FIR, order 220). Then, we compared the ERPs from both conditions using a cluster-based dependent samples t-test with similar parameters as before, only the cluster alpha criterion decreased to 0.01 for a more focal cluster. Next, we calculated absolute amplitude differences, averaged over the resulting negative cluster for each participant. Similarly, we calculated absolute differences of normalized oscillatory power between *illusion* and *no-illusion* trials (averaged over the significant cluster identified from modeling). Finally, we correlated these differences of amplitude and power across participants.

## Supplementary information

Figure S1

## Data Availability

Raw data cannot be made available because participants did not consent to public dissemination. Processed data can be made available upon reasonable request.
