# Supplementary material for: Single trial prestimulus oscillations predict perception of the sound-induced flash illusion
Source: Sci Rep. 2019 Apr 12;9:5983. doi: 10.1038/s41598-019-42380-x (PMC6461663; doi:10.1038/s41598-019-42380-x)
Supplement: Supplementary file 1 — Figure S1 [file 41598_2019_42380_MOESM1_ESM.pdf]

Supplementary Information for:

Single trial prestimulus oscillations predict perception of the sound-induced flash illusion

Mathis Kaiser, Daniel Senkowski, Niko A. Busch, Johanna Balz, Julian Keil

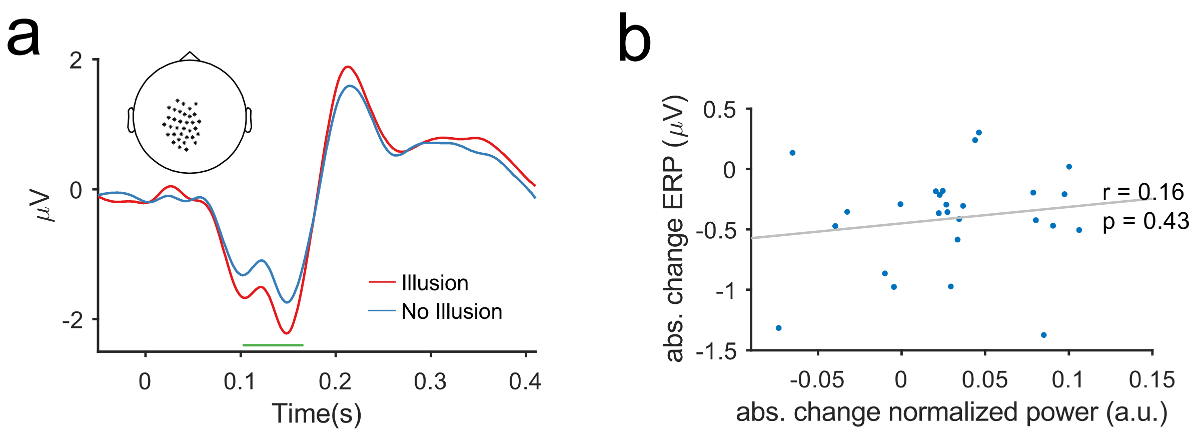

Supplementary figure S1

a: Event-related potentials for illusion and no illusion trials, averaged over channels with significant differences between conditions. A green line above the abscissa indicates time intervals with significant differences. Asterisks in the channel layout indicate channels with significant differences.

b: Pearson's correlation between the absolute change of normalized power, averaged over the significant cluster obtained from the modeling analysis, and absolute change of ERP amplitudes, averaged over the significantly different cluster indicated in panel *a*.
